# A data driven machine learning approach to differentiate between autism spectrum disorder and attention-deficit/hyperactivity disorder based on the best-practice diagnostic instruments for autism

**DOI:** 10.1038/s41598-022-21719-x

**Published:** 2022-11-05

**Authors:** Nicole Wolff, Gregor Kohls, Judith T. Mack, Amirali Vahid, Erik M. Elster, Sanna Stroth, Luise Poustka, Charlotte Kuepper, Stefan Roepke, Inge Kamp-Becker, Veit Roessner

**Affiliations:** 1 Department of Child and Adolescent Psychiatry and Psychotherapy, Faculty of Medicine, Technische Universität Dresden, Dresden, Germany; 2 Department of Child and Adolescent Psychiatry, Psychosomatics and Psychotherapy, Medical Clinic, Philipps-University Marburg, Marburg, Germany; 3 Department of Child and Adolescent Psychiatry/Psychotherapy, University Medical Center Göttingen, Göttingen, Germany; 4 Institute of Psychology, Humboldt-Universität zu Berlin, Berlin, Germany; 5 Department of Psychiatry, Campus Benjamin Franklin, Charité - Universitätsmedizin Berlin, Berlin, Germany; 6 Institute and Policlinic of Occupational and Social Medicine, Faculty of Medicine, Technische Universität Dresden, Dresden, Germany

**Keywords:** Experimental models of disease, Autism spectrum disorders, ADHD

## Abstract

Autism spectrum disorder (ASD) and attention-deficit/hyperactivity disorder (ADHD) are two frequently co-occurring neurodevelopmental conditions that share certain symptomatology, including social difficulties. This presents practitioners with challenging (differential) diagnostic considerations, particularly in clinically more complex cases with co-occurring ASD and ADHD. Therefore, the primary aim of the current study was to apply a data-driven machine learning approach (support vector machine) to determine whether and which items from the best-practice clinical instruments for diagnosing ASD (ADOS, ADI-R) would best differentiate between four groups of individuals referred to specialized ASD clinics (i.e., ASD, ADHD, ASD + ADHD, ND = no diagnosis). We found that a subset of five features from both ADOS (clinical observation) and ADI-R (parental interview) reliably differentiated between ASD groups (ASD & ASD + ADHD) and non-ASD groups (ADHD & ND), and these features corresponded to the social-communication but also restrictive and repetitive behavior domains. In conclusion, the results of the current study support the idea that detecting ASD in individuals with suspected signs of the diagnosis, including those with co-occurring ADHD, is possible with considerably fewer items relative to the original ADOS/2 and ADI-R algorithms (i.e., 92% item reduction) while preserving relatively high diagnostic accuracy. Clinical implications and study limitations are discussed.

## Introduction

Autism spectrum disorder (ASD) and attention-deficit/hyperactivity disorder (ADHD) are two of the most commonly diagnosed neurodevelopmental conditions in childhood and adolescence^1^. Individuals with ASD show restricted and repetitive behaviors, persistent impairments in their social communication and interaction abilities and may also report sensory issues^2^. Typical ADHD symptoms entail impulsivity, hyperactivity, and inattention that cause serious functional impairments across different social settings^3^. While there is evidence to support the view that ASD and ADHD are separate mental health conditions, they appear to be closely related^4^. In fact, research suggests that diagnoses of ASD and ADHD frequently co-occur^5,6^. This points to considerable overlap at both the clinical and etiopathophysiological level, presenting for instance practitioners with challenging differential diagnostic considerations^7^. In particular, the correct classification of an individual’s clinical symptoms as ASD- and/or ADHD-specific is pivotal for a precise diagnosis, and this in turn is important for patients and their families in terms of adequate healthcare and rights for compensation and social support (e.g., financial aid), which differ depending on the respective diagnosis/diagnoses^8^. Consequently, investigating the phenotypic boundaries and commonalities between both conditions has become a timely and urgent topic^5,6,9^.

To date, research has shown that children with ADHD often have social difficulties that may resemble those seen in ASD, including problems in relating to other people or inappropriate peer-related behaviors^10–12^. Similarly, core symptoms of ADHD, like inattention and hyperactivity, are often found also in children with ASD^13^. However, the extent to which the two conditions clearly share difficulties remains poorly understood. This is partly owed to the fact that the vast majority of relevant work is based on caregiver questionnaires or on broadband psychopathology screening measures that are rather unspecific diagnostically. More recently, though, different research groups have started to use best-practice diagnostic instruments for ASD to explore more thoroughly ASD-like impairments in ADHD, and to tests the validity of these measures to differentiate clinically between ASD and ADHD (e.g., Refs.^14,15^).

There exist two widely used and scientifically established instruments developed specifically to evaluate ASD symptoms. One is the Autism Diagnostic Observation Schedule (ADOS) which is a direct clinical observation assessment^16^, and the second is the Autism Diagnostic Interview-Revised (ADI-R) which is a parental interview^17^. With respect to the ADOS, good sensitivity and specificity has been demonstrated in research settings when analyzing individuals with “pure” ASD vs. those without any diagnosis (ND), while relatively little is known about its utility and accuracy in youth and adults with co-occurring psychiatric disorders^18^. Recently, Colombi et al. analyzed data from a clinical sample with a suspected ASD diagnosis that was assessed with the ADOS-2 modules 3 or 4 (M3/M4: indicating verbal fluency and age). Individuals of this sample where initially diagnosed with one of the following diagnoses: psychotic disorder, mood disorder, or disruptive disorder (e.g., conduct disorder) but not ASD. Individuals were admitted consecutively to the psychiatric inpatient unit for acute psychiatric symptoms suspected of having ASD. Patients with a previous diagnosis of ASD were excluded. The study found generally low sensitivity and specificity in detecting/excluding ASD for both M3 (sensitivity: 58.3%, specificity: 56.5%) and M4 (sensitivity: 55.6% and specificity: 59.5%). Therefore, the authors recommended caution when interpreting the results of the ADOS-2 in psychiatric individuals with a consisting (comorbid) diagnosis and a following ASD suspicion. However, they pointed out that their findings are limited because the study was conducted at a single site and with a limited number of participants. They recommended that the results ideally be replicated and extended with a larger sample from different sites using the two best-practice diagnostic tools for ASD, ADOS/2 and ADI-R^19^.

Interestingly, a recent review and meta-analysis^20^ of the accuracy of the ADOS-2 and the ADI-R in clinical compared to research settings found that the ADOS-2 is more accurate than the ADI-R in detecting ASD, and it was confirmed that sensitivity and specificity are less accurate in (retrospective) clinical analysis than in “real time” research settings, with specific in- and exclusion criteria. Therefore, the authors concluded that more research in clinical populations, in particular, is urgently needed. Using both ADOS (i.e., the predecessor version of the ADOS-2) and ADI-R, Grzadzinski et al.^14^ investigated a clinically referred group of children who received best-estimate clinical (BEC) diagnoses. They focused on the presence of either ASD or ADHD. First, they compared the proportion of children in both groups who met standard ADOS and ADI-R cut-offs for an ASD diagnosis. The ADOS cut-off was met by 85% of the children with a BEC of ASD and by 21% of the children with a BEC of ADHD, while the ADI-R cut-offs were met by 67% of the children with a BEC of ASD and by 30% of the children with a BEC of ADHD. The authors then focused on ASD symptoms that most adequately differentiated between the two conditions by only including symptoms which were endorsed in ≥ 66% of the group with a BEC of ASD and ≤ 33% of the group with a BEC of ADHD (note that “restricted and repetitive behavior” items were not considered because endorsement was expected to be low). There were four ADOS items from the social communication domain (i.e., amount of reciprocal social communication, quality of social overtures, unusual eye contact, and facial expressions directed to others), but none of the ADI-R items, that met this criterion for adequate discrimination.

The results of the Grzadzinski study clearly highlight the challenge of practitioners to distinguish a child with ASD from one with ADHD in clinical settings even using best-practice instruments. While differences in the type and quality of social impairments between ASD and ADHD seem to exist^21^, children with ADHD however display a large amount of ASD-like symptoms—certainly those individuals who are clinically referred due to ASD concerns, underscoring the fact that social difficulties are also common in many individuals with ADHD^22^.

Notably, the above-mentioned study did not include children with a co-occurring ASD + ADHD. Thus, it remains unclear to what extent the two best-practice diagnostic instruments in the field of ASD are actually able to reliably distinguishing between individuals with ASD or ADHD versus those with the co-occurrence of both conditions. Considering that ASD + ADHD may qualify as a potentially distinct subtype compared to the two single diagnoses (“overarching disorder hypothesis”^23–25^), it is critically and clinically relevant to know whether and which specific symptom sets best characterize and separate not only both pure groups, but all three groups. A better understanding of such phenotypic differences and similarities could inform clinical interventions that are more targeted^6,26,27^.

Recently, clinical research has begun to use machine learning (ML) methods to identify useful phenotypic information from the ADOS^28–30^, the ADI-R^31,32^, both instruments in combination^33^, or other sources, such as home videos^34^, attempting to improve the decision-making process for screening and/or diagnosing individuals with ASD versus those without ASD^35–37^. ML techniques allow researchers to ideally detect reduced subsets of diagnostic features out of these usually multi-hour long clinical examinations necessary for making a diagnosis. ML studies suggest that detecting ASD can be achieved with considerably fewer items relative to the original ADOS and ADI-R algorithms while preserving high diagnostic accuracy (Ref.^29^; but see^38^ for a critical view on current trends in the assessment of ASD).

To our knowledge, Duda et al.^39^ were the first to apply ML algorithms to distinguish ASD from ADHD by using the 65-item Social Responsiveness Scale (SRS), which is a parent-reported screening questionnaire on the severity of autistic characteristics^40^. This study found that five behaviors measured by the SRS (e.g., “Trouble with the flow of normal conversation”) were sufficient to distinguish ASD from ADHD with high accuracy (area under the curve, AUC = 0.965). However, Duda et al.^39^ did not apply ML on either ADOS or ADI-R, and—like Grzadzinski et al.^14^—they did not include individuals with co-occurring ASD + ADHD or those without any clinical diagnosis.

Thus, the primary aim of the current study was to use ML (i.e., support vector machine/SVM) in order to determine whether and which ADOS and ADI-R items would be most informative to separate between individuals with BEC of ASD, ADHD and those with ASD + ADHD versus individuals with no psychiatric diagnosis (ND) who were all referred to specialized clinics due to ASD concerns. We chose SVM because SVMs have several advantages over other ML algorithms (Ref.^41^, see also “Methods” section), and according to a recent meta-analysis^42^, SVM is the most accurate and widely used classifier in distinguishing individuals with ASD from those without ASD, ensuring the likelihood that results can be replicated (see Ref.^43^) for a discussion of this issue in the context of ML). Since most relevant ML work has been conducted in English-speaking countries (primarily the US), we aimed to extend this line of research with a large and diverse multi-site German sample to increase generalizability of the analyses and findings, and thus improve our international understanding of the differential diagnosis of ASD vs. ADHD using ML techniques. Moreover, we focused on a dataset that included many individuals with the BEC diagnosis of ASD + ADHD to challenge the performance of the ML algorithm in light of the particularly difficult nature to differentiate patients with ASD from those with co-occurring ADHD by using the best-practice diagnostic instruments for autism. Our main a priori hypothesis was to find specific behaviors particularly in the social domain, such as difficulties in social communication, that best differentiate between ASD (incl. ASD + ADHD) and ADHD, but also between ASD (incl. ASD + ADHD) and ND. Being able to identify behavioral features that distinguish between the groups may help developing novel training and screening tools for differential diagnostics based on the identified set of features.

## Results

### Specificity and sensitivity of ADOS and ADI-R total cut-offs across groups

76% of the ASD group and 68% of ASD + ADHD group met the ADOS/2 total cut-offs for ASD (Table 1). With respect to the ADI-R, only 45% of the ASD group and 44% of the ASD + ADHD group met all three domain cut-off scores. Combining both instruments revealed that 33% of the ASD group and 32% of the ASD + ADHD group met all cut-offs for ASD, indicating a relatively high false negative rate. By contrast, 100% of the ADHD group and 96% of the ND group did not meet ASD cut-offs when both instruments were considered together (i.e., high true negative rates; see Table 1).

**Table 1:** Distribution of individuals per group that met total cut-off scores for ASD on the ADOS and/or ADI-R.

Instruments/modules	ASD	ASD + ADHD	ADHD	ND	Statistical comparison
	True positive rate: sensitivity in % and n of children who meet the cut-off score for ASD		True negative rate: specificity in % and n of children who do not meet the cut-off score for ASD		
ADOS M3	73.6% (356)	68.1% (64)	88.2% (135)	85.3% (267)	*Χ*^2^ (3) = 361.59, *p* < 0.001
ADOS M4	77.0% (414)	66.7% (17)	89.6% (146)	84.9% (281)	*Χ*^2^ (3) = 361.59, *p* < 0.001
ADI-R	44.5% (181)	44.4% (32)	83.3% (55)	91.7% (154)	*Χ*^2^ (3) = 430.24, *p* < 0.001
ADOS M3 and ADI-R combined	32.5% (111)	31.7% (19)	100% (65)	97.3% (146)	*Χ*^2^ (3) = 76.74, *p* < 0.001
ADOS M4 and ADI-R combined	35.4% (137)	28.1% (18)	100% (66)	96.9% (154)	*Χ*^2^ (3) = 87.72, *p* < 0.001

The table depicts the obtained true positive rates as well as the true negative rates of the four groups with respect to the Autism Diagnostic Observation Schedule (ADOS), the Autism Diagnostic Interview-Revised (ADI-R), and both instruments combined.

*ASD* autism spectrum disorder, *ADHD* attention-deficit/hyperactivity disorder, *ND* no psychiatric diagnosis.

### SVM with and without feature selection

For each of the six group comparisons, we ran SVM first without feature selection, followed by SVM with feature selection focusing on the five most important features that discriminated best between groups. As shown in Table 2, the all-feature model performed excellently (AUCs ≥ 0.8) in discriminating between ASD vs ND (and ASD + ADHD vs ND) as well as between ADHD vs. ASD (and ADHD vs. ASD + ADHD). The discrimination of ADHD vs ND and of ASD vs. ASD + ADHD was poor (AUCs ≤ 0.7; see Table 3).

**Table 2:** Performance of the SVM machine learning algorithm in discriminating between the different group constellations.

	Group differentiation					
	ND vs. ASD	ND vs. ASD + ADHD	ADHD vs. ASD	ADHD vs. ASD + ADHD	ND vs. ADHD	ASD vs. ASD + ADHD
All-feature model: AUC (Sensitivity, Specificity)	0.91 (0.90, 0.88)	0.86 (0.41, 0.72)	0.91 (0.68, 0.73)	0.84 (0.89, 0.78)	0.62 (0.04, 0.39)	0.42 (0, 0)
Five-feature model: AUC (Sensitivity, Specificity)	0.77 (0.88, 0.81)	0.86 (0.51, 0.78)	0.91 (0.73, 0.82)	0.89 (0.90, 0.87)	0.40 (0, 0)	0.48 (0, 0)
DeLong test (all-feature vs. five-feature model): *p*-value	< 0.001	*ns*	*ns*	0.007	< 0.001	*ns*
Five most discriminative ADOS/ADI features (rank ordered):	Facial expressions directed to others Amount of reciprocal social communication Unusual eye contact Imaginative play (ADI) Hand and finger mannerisms (ADI)	Unusual eye contact Head shaking (ADI) Hand, finger and other complex mannerisms Shared enjoyment in interactions Compulsions/Rituals (ADI)	Facial expressions directed to others Stereotyped/ idiosyncratic use of words or phrases Amount of reciprocal social communication Conversation Friendships (ADI)	Quality of social overtures Response to approaches of other children (ADI) Descriptive, conventional, instrumental or informational gestures Hand and finger mannerisms (ADI) Conversation	Overactivity Abnormal development at or before 36 months (ADI) Tantrums, aggression, negative or disruptive behavior Offering comfort (ADI) Amount of reciprocal social communication	Asks for information Offers information Pronominal reversal (ADI) Language production and linked nonverbal communication Insight

Additionally, a test with a sensitivity (i.e., positive test result in presence of the condition) and specificity (i.e., negative test result in absence of the condition) of around 75% to 90% can be considered to have reasonable to good diagnostic performance.

*ND* no psychiatric diagnosis, *ASD* autism spectrum disorder, *ADHD* attention-deficit/hyperactivity disorder, *ns* not significant; *AUC* area under the curve: AUC < 0.7 (poor discrimination), 0.7 ≤ AUC < 0.8 (acceptable discrimination), 0.8 ≤ AUC < 0.9 (excellent discrimination), AUC ≥ 0.9 (outstanding discrimination) according to Hosmer et al. .^44^.

**Table 3:** Main sample demographics.

	ASD (*n* = 574)	ADHD (*n* = 164)	ASD + ADHD (*n* = 113)	ND (n = 344)	Group comparisons
**Age (in years)**					
M (SD)	15.8 (± 10.0)	10.7 (± 4.3)	11.5 (± 3.2)	16.1 (± 12.0)	*F*(3, 1183) = 18.41, *p* < 0.001
min–max	5–72	5–49	5–18	5–62	
**Gender**					
Female	15%	7%	11%	18%	*Χ*^2^(*df* = 3) = 11.21, *p* = 0.011
Male	85%	93%	89%	82%	
**IQ**^#^					
*n*	486	139	91	267	*F*(3, 982) = 1.70, *ns*
M (SD)	101.1 (± 18.5)	97.8 (± 20.6)	97.5 (± 19.7)	99.5 (± 19.9)	
Min–max	55–145	62–138	58–132	58–136	

*ASD* autism spectrum disorder, *ADHD* attention-deficit/hyperactivity disorder, *ASD* + *ADHD* co-occurrence of both diagnoses, *ND* no psychiatric diagnosis, *N/A* not applicable, *ns* not significant;

^#^Considering solely an IQ criterion of < 70 as a cut-off for Intellectual Disability (ID), there was the following proportion of ID per group: ADHD (1.9%), ASD (2.2%), ASD + ADHD (4.3%), and ND (4.7%), with no significant differences between groups (p = 0.15).

When considering the five most discriminative features for each of the six group comparisons (features are listed in Table 2), SVM with five-feature selection performed significantly worse for ASD vs ND and for ADHD vs ND, slightly but significantly better for ADHD vs. ASD + ADHD, with no performance differences observed for the three remaining group contrasts. Regarding the latter finding, this suggests that both SVM models had a similar accuracy in predicting group status. In contrast, model accuracy in distinguishing ASD from ND was substantially lower, but still in the acceptable range, when only the reduced subset of the five highest ranking behavioral features were included. Across all group comparisons that involved an ASD group (ASD & ASD + ADHD) relative to a non-ASD group (ADHD & ND), the five most distinguishable features stemmed from both ADOS (i.e., clinical observation) and ADI-R (i.e., parental interview), and they corresponded to the social-communication but also restrictive and repetitive behavior domains. Notably and not unexpectedly, several of the most discriminative features for ADHD vs ND appeared to be ADHD-related (e.g., overactivity), which were however accompanied by social difficulties (e.g., social communication deficits); overall model performances in differentiating between the two groups were very poor though.

## Discussion

The primary aim of the current study was to determine whether and which ADOS and ADI-R items would be most informative to separate between the four groups of individuals referred to specialized ASD outpatient clinics (i.e., ASD, ADHD, ASD + ADHD, ND). We first compared the percentage of individuals in each group who met clinical cut-offs for ASD on the ADOS and/or ADI-R, which was intended to replicate and extend the findings by Grzadzinski et al.^14^ who did not investigate individuals with co-occurring ASD + ADHD or those without any psychiatric diagnoses. We observed that 76% of the individuals with ASD (as compared to 85% in Grzadzinski’s work), and 11% of the individuals with ADHD (as compared to 21%) met ADOS/2 cut-offs for ASD. Additionally, and novel, 68% of the ASD + ADHD group and 15% of the ND group met the clinical cut-off on the ADOS. With respect to the ADI-R, 45% of the ASD group and 44% of the ASD + ADHD group in contrast to 17% of the individuals with ADHD and 8% of the individuals with ND met all three domain cut-offs. Finally, while combining both ADOS and ADI-R resulted in excellent specificity rates for individuals with ND and those with ADHD (between 97 and 100%), sensitivity rates ranged low between 30 and 34% for both ASD groups. Thus, using a combination of ADOS and ADI-R diagnostic cut-off scores decreased substantially the risk of false positives. However, it increased dramatically the risk of false negatives, for instance, as compared to the single usage of the ADOS. This has implications for clinical practice, including specialized ASD clinics that deal with a great number of referrals due to ASD concerns who eventually end up not having ASD but another psychiatric condition requiring proper assessment and treatment, or who have autistic characteristics without any psychiatric diagnosis. Our finding that ~ 65% of individuals with a BEC diagnosis of ASD did not reach the clinical cut-offs on either ADOS and ADI-R emphasizes that (i) ASD cannot simply be diagnosed by schematically applying cut-off scores alone, but (ii) additional clinical information from a patient’s medical record (and potentially other sources) need to be gathered to confirm the diagnosis in conjunction with professional clinical judgement.

The main innovative aspect of the current study was that we applied an independent and data driven machine learning approach using SVM to determine whether and which ADOS and ADI-R items would best differentiate within the clinical sample at hand. Here we focused on the five most important discriminative features (vs. the entire set of behavioral features provided by ADOS/ADI-R). We found that a reduced subset of five features from both ADOS and ADI-R reliably differentiated between non-ASD and ASD groups, ranging from acceptable (ASD vs ND) to excellent (ADHD/ND vs. ASD + ADHD; ADHD vs. ASD) discrimination. Overall, our findings support earlier ML studies showing a limited number of crucial features contribute to classificatory precision that is just as accurate as leveraging diagnostic information from the full examination of ADOS and/or ADI-R. Notably, though, when comparing ASD with ND, the five-feature model performed worse than the all-feature model in separating both groups, suggesting a disadvantage of the former over the latter regarding its classification accuracy (AUC: 0.77 vs. 0.91). This is in contrast to the findings by Kamp-Becker and colleagues (2021) who used the random forest algorithm on ADOS and ADI-R combined and reported similar classification rates for full-feature and reduced-feature models when comparing ASD with a non-ASD group. However, their sample differed from ours as it included a large number of individuals with a wide range of different co-occurring psychopathologies, while the current sample had no other psychiatric diagnoses in addition to the primary BEC (ASD and/or ADHD) (or the lack thereof) assigned to the four groups tested here. This likely explains the different findings.

It should be emphasized, though, that our five-feature classifier was still sufficient enough to reliably distinguish ASD from ND with 88% sensitivity and 81% specificity, which is higher than classificatory performance via the combination of conventional ADOS and ADI-R cut-off scores. Thus, it appears conceivable that the clinical assessment, including the screening for ASD, could be made a less complex endeavor by drawing clinicians’ attention to the most specific behavioral features of ASD in order to help in the (differential) diagnostic process. In the long term, this can facilitate decision-making with regard to whether a patient with signs of ASD actually needs a comprehensive—usually multi-hour—standard clinical evaluation for ASD or not.

The five most relevant items selected by the SVM algorithm to differentiate ASD (and ASD + ADHD) from ND, partly confirm our predictions. We indeed found that the majority of discriminative features belonged to the social domain, but several restricted and repetitive behavioral features were picked up by the algorithm, too, and stemmed from both ADOS and ADI-R. Notably, all identified features in the current study are also part of the clinical scoring algorithms as proposed by the ADOS/2 and ADI-R manuals which underscores their diagnostic utility. Two of these behavioral features were ranked among the most relevant ones for both ASD groups compared to ND, including unusual eye contact, and hand/finger mannerisms. If replicated in independent samples, this finding suggests that – irrespective of co-occurring ADHD—such behaviors (singly or in conjunction with other features, such as listed in Table 3) might be the most informative ones in differentiating individuals with ASD from those without a formal psychiatric diagnosis but with ASD-like characteristics (i.e., having signs of ASD). Particularly in the context of specialized institutions, these behaviors can be helpful as preclinical tools in detecting a true increased likelihood of full-fledged ASD.

Using SVM to compare ASD with ADHD, we found excellent classification accuracies for both the full-feature (AUC ≥ 0.91, sensitivity ≥ 68%, specificity ≥ 73%) and the five-feature model (AUCs ≥ 0.91, sensitivity ≥ 73%, specificity ≥ 82%). These results corroborate and extend the ML findings by Duda et al.^39^ who used the SRS questionnaire showing that a reduced subset of only 5 (vs. 65) screening items had an excellent accuracy for distinguishing between the two conditions. In fact, similar to the Duda study, we also found that predominantly social (e.g., peer relationship problems) but also restrictive and repetitive behaviors (e.g., stereotyped language use) were among the most valuable classifiers in this regard. This pertained to both group comparisons—ASD vs. ADHD and ASD + ADHD vs. ADHD, while the latter one is a novel addition to the existing literature. Interestingly, “trouble with the flow of normal conversation” was the highest-ranking feature in the work by Duda et al.^39^. Although “conversation” (ADOS item A8) was not the highest-ranking feature in the current study when comparing ASD or ASD + ADHD with ADHD, it was the one that overlapped across both group comparisons, indicating its promise for evaluating the risk of ASD—irrespective of co-occurring ADHD—relative to pure ADHD. Notably, “conversation” was also the ADOS item with the highest importance value to accurately classify ASD vs. mood and anxiety disorder (MAD) in a recent study by Ref.^45^. In fact, five out of the eight items (i.e., 5 ADOS items, and 3 ADI-R items) listed in the Wittkopf study as the ones with the greatest classification significance were also selected by the SVM algorithm used here in the ASD versus ADHD group comparisons: conversation, facial expressions directed to others, stereotyped/ idiosyncratic use of words or phrases, amount of reciprocal social communication, and quality of social overtures. Note, these are all exclusively ADOS items, similar to the results reported by Grzadzinski et al.^14^. Taken together, these cross-study findings suggest that the presence of a minimal subset of observable behaviors, particularly in the social interaction and communication domains, appear to be ASD-specific in distinction to other often co-occurring mental health conditions, such as ADHD or MAD.

Remarkably, Grzadzinski et al.^14^ did not analyze ADOS and ADI-R items tapping into restricted and repetitive behavior when comparing ASD with ADHD because the authors expected endorsement to be negligible. Our findings (but also the Duda study) do not support this assumption. Although, as expected, the vast majority of features that best classified ASD relative to ADHD stemmed from the social interaction and communication domains (as outlined above), our ML algorithm also identified two items from the restricted and repetitive behavior domain, including stereotyped/ idiosyncratic use of words or phrases, and hand/finger mannerisms. These data may suggest that this behavioral domain is in fact less relevant for differentiating ASD from ADHD. At the same time, it may also signify that restricted and repetitive behavior items are generally less reliable than social-communication ones when coding them (e.g., indicated by lower interrater reliabilities^46^). One reason for such reduced objectivity might be the lower occurrence frequency of these behaviors which hampers their valid identification (e.g., Ref.^47^). Therefore, one could conclude to date that they are of limited usability in discriminating ASD from ADHD, or other mental health conditions such as MAD^45^.

In contrast to the studies by Duda et al.^14^ and Grzadzinski et al.^39^, we did not find “unusual eye contact” to be among the five highest ranking classifiers that differentiated ASD and ADHD. But we found that this particular behavioral feature separated well ASD (both groups) from the ND group (see above). This suggests that—in addition to several other ASD-related symptoms—atypical eye contact might be a behavioral feature that overlaps considerably in ASD and ADHD, at least in this clinically-referred sample (see for similar findings, e.g., Ref.^48^). Therefore, the presence of abnormal eye contact appears to be rather ASD-unspecific as it does not adequately discriminate ASD from ADHD (or ASD from MAD^45^).

Unsurprisingly, the SVM algorithm performed poor in discriminating between ND vs. ADHD as well as between ASD vs. ASD + ADHD (AUCs ≤ 0.62). Interestingly, with regard to ND vs. ADHD, the algorithm picked several discriminative features that are apparently ADHD-related, such as overactivity, or disruptive behaviors. However, considering the inadequate classification accuracy of the five-feature model that identified these features, this finding lacks reliability and thus should not be further interpreted.

Concerning the ASD vs. ASD + ADHD comparison, our data would not support the notion that the group of individuals with ASD + ADHD qualify as a potentially distinct subgroup than ASD without ADHD. Both groups were diagnostically quite indistinguishable when the current best-practice assessment instruments, ADOS and ADI-R, are administered and analyzed with machine learning. This corroborates our prior findings using ‘traditional’ variance analyses on the ADOS/ADI-R in form of a full-factorial group design (Ref.^21^; see also^49^ who found no phenotypic differences between ASD and ASD + ADHD when measured with ADOS and ADI-R).

The findings of the current study need to be interpreted in light of a few limitations. First, the entire sample, including the individuals without a psychiatric diagnosis (ND group) as well as those with ADHD without ASD, was recruited via specialized ASD clinics in Germany. This may have biased our results to some extent because both groups, ND and ADHD, were pre-selected due to ASD concerns and thus are not fully representative subsamples of their respective population. This selection bias may also have resulted in the two ADHD groups being slightly younger and having a higher male-to-female ratio than the two other groups (i.e., ND and ASD). Nevertheless, the sample as a whole is unique as it stems from a clinical rather than a traditional research population, representing a ‘typical’ population in German outpatient clinics for ASD which is most relevant to practitioners (e.g., cases typically referred to ASD clinics cause most differential diagnostic difficulties due to overlap in symptomatology). Note, though, that we deliberately excluded nonverbal individuals and those with other co-occurring psychiatric diagnoses, thereby limiting the generalizability of our findings to the entire ASD population. To advance this line of research forward, it would be ideal to recruit and analyze clinical (and/or research) samples across different countries that are not limited to specialized ASD clinics, but also include other data sources to replicate the results obtained and thus improve the interpretation and generalization of the current findings. This could also entail a comparison of different ML algorithms, as done by Ref.^39^, to better understand which algorithms (and their specifications) are more capable of detecting specific features for differential diagnostic purposes. Second, we analyzed the diagnostic data from children, adolescents, and adults combined. While certain ASD-related behaviors appear to be stable over the life course, there is also evidence to suggest that ASD symptom presentation may change from child- into adulthood with general improvements in symptomatology with age^50^. However, we intentionally decided to not split and compare the four groups by specific age bands due to limited statistical power. Note that we already used the SVM algorithm on six group comparisons. Thus, splitting the sample into for instance two age bands, children/adolescents versus adults, would have inflated our analyses to 12 comparisons rendering a proper interpretation nearly impossible. Moreover, we were only able to include relatively few older individuals with ADHD. Hence, dividing the sample by age would have led to unmanageably small subgroups to compare. However, it would be interesting to see how our machine learning approach performs in adequately powered groups of youth versus adults.

Finally, we restricted our SVM analyses to select only the five most discriminable features per group comparison. Although one can argue that this number is rather arbitrary, it corresponds to the smallest number of ADOS/ADI-R features previously identified by different machine learning algorithms when comparing ASD with non-ASD groups. Therefore, the present approach may facilitate the comparability of findings reported in other relevant work current and in the future.

In conclusion, the results of the present study support the idea that detecting ASD in individuals with suspected signs of the diagnosis, including those clinically more complex cases with co-occurring ADHD, is possible with considerably fewer items relative to the original ADOS/2 and ADI-R algorithms (i.e., 92% item reduction) while preserving relatively high diagnostic accuracy (but see^38^ for a critical view on current trends in the assessment of ASD, including the shortening of the diagnostic process). We certainly acknowledge that further studies in independent samples are warranted to further determine the clinical utility of the identified diagnostic classifiers. If replicated, these results may benefit the development of novel practitioner-oriented training tools to detect ASD (vs. other conditions) more efficiently and/or optimize the screening, triaging and diagnosing of those individuals seen in specialized institutions by aiding clinical experts particularly in the challenging process of differential diagnosis. Notably, individuals who are referred to ASD outpatient clinics are usually not evaluated for ADHD as part of the assessment routine due to numerous pragmatic considerations (e.g., personnel and financial resources, or time constraints of patients and their families). Thus, one could contemplate the feasibility of developing some kind of ADHD screening score based the behavioral classifiers from ADOS/ADI-R that best separated ADHD from the other groups. This could guide the decision-making process of whether an additional evaluation for ADHD is clinically justified or not.

## Methods

### Sample

The sample was derived from Germany’s largest database of individuals referred to specialized ASD outpatient clinics^51^. The whole database includes 2453 individuals (16.8% female; age: 1–72 years, *M* = 13.56 ± 10.61) of whom 1260 (51.4%) were diagnosed with ASD, others had another mental condition (n = 844; 34.4%; e.g., ADHD, mood or anxiety disorders), and 349 (14.2%) did not receive any psychiatric diagnosis. All individuals were diagnosed according to the International Classification of Diseases ICD-10^52^ using “gold standard” best estimate clinical (BEC) diagnoses^53^. Any BEC diagnosis was determined by at least two experienced clinicians from a multidisciplinary team (incl. psychologists and/or psychiatrists) after extensive examination and review of all available information from a patient’s medical record that included—amongst others—IQ test results, the Autism Diagnostic Observation Schedule (ADOS^16^, the Autism Diagnostic Interview-Revised (ADI-R^17^, and a differential/co-occurring diagnoses algorithm performed by an experienced psychiatrist. ADOS and ADI-R were conducted by clinically trained team members at each center who were all licensed to do so. The study was approved by the ethics committee of the Philipps-University Marburg (Az. 92/20). Due to the retrospective nature of our data collection and analysis, the need for informed consent was waived by the ethics committee. All methods were performed in accordance with the relevant guidelines and regulations.

For the purpose of the present study, our sample was selected according to the following criteria: (i) referred for a clinical ASD diagnostic assessment, (ii) complete data of ADOS Module 3 or Module 4, and ADI-R, (iii) verbally fluent, (iv) BEC diagnosis of ASD (F84.0, F84.1, or F84.5) but no ADHD, (v) BEC diagnosis of ADHD (all subtypes F90.0 or F98.8) but no ASD, (vi) co-occurring ASD + ADHD, and (vii) no psychiatric diagnosis (ND). The patients with ASD, ADHD and ASD + ADHD had no other psychiatric diagnoses. With respect to the ADOS, we focused on modules M3 and M4 indicating verbal fluency, because differentiation between ASD and ADHD is especially challenging in verbally fluent patients with average intellectual functioning^14,54,55^. This resulted in a final sample of n = 1195 individuals (age in years: M = 14.8 ± 9.9, min = 5, max = 72; 14.5% female), including n = 574 individuals with ASD, n = 164 with ADHD, n = 113 with ASD + ADHD, and n = 344 with ND (Table 3). ADOS M3 and M4 data were available from 66.3 and 33.7% of the included participants, respectively. Here, we chose to analyze M3 and M4 data combined in order to be comparable to earlier relevant research^14,55^. Please note that the present data are based on the ADOS, but not the more recent ADOS-2 manual^56^. This is because retrospective data collection spanned across a relatively long time period, dating back to when the German version of the ADOS-2 was not yet available.

### Diagnostic measures and analytic strategy, including machine learning

The current analyses included the item scores of the so-called “gold standard” or best-estimate instruments in diagnosing ASD^57^: ADOS^16,58^ and ADI-R^59,60^. Both are based on ASD criteria of ICD-10^52^ and DSM-IV-TR^61^, and they can be used to obtain information about ASD symptoms across different behavioral domains. More details on these two well-established measures can be found in the Supplement.

First, we calculated the proportion of individuals in each of the four groups who met diagnostic algorithm cut-offs in both ADOS and ADI-R, following the analytic procedure analogous to Ref.^14^. We applied the following cut-offs for the ADOS/2 (M3: total cut-off spectrum ≥ 8; M4: total cut-off spectrum ≥ 7). According to the ADI-R manual, no total cut-off is to be calculated. However, for the current analysis, we defined the criteria, like Grzadzinski et al.^14^, that patients who met ASD cut-offs on the three ADI-R domains (“communication” ≥ 10, “social interaction” ≥ 7, “RRB” ≥ 3) reached the ADI-R total cut-off.

We then used support vector machine (SVM) analyses to evaluate which ADOS and ADI-R items are able to discriminate best between ASD (incl. ASD + ADHD) and ADHD, but also between ASD (incl. ASD + ADHD) and ND. SVM is a robust machine learning algorithm, which can be used to examine data for various purposes, such as classification or regression analyses, in order to solve big data classification problems^41^. SVM is used to find a hyperplane with the maximum margin (i.e., distance between data points from distinct classes) in an n-dimensional space (where n is the number of features) to differentiate between classes^62^. For instance, SVM has been shown to perform with high accuracy particularly in distinguishing between ASD and ADHD by utilizing only 5 screening items from the SRS^39^. SVM is the one algorithm that is most frequently used in this line of research likely due to its high predictive power for ASD classification^37^. According to a recent meta-analysis^42^, SVM is the most accurate classifier in distinguishing individuals with ASD from those without ASD, ensuring the likelihood that results can be replicated (see^43^ for a discussion of this issue in the context of the use of ML for ASD diagnostics). SVMs retain several attractive properties: They can deal with noisy, highly correlated features and high-dimensional data sets, and they are resistant to overfitting and thus generalize well^41^.

We adopted a binary classification approach, i.e., we examined pairs of diagnostic groups, resulting in six possible combinations (see Table 2). Our SVM approach for each combination consisted of two steps: First, we ran SVM without feature selection. Second, we applied feature selection to identify the five most relevant ADOS and/or ADI-R features that best discriminated between groups, consistent with recent findings^29,31,39^. We then tested whether the two approaches differed significantly from each other to validate the five-feature model versus the all-feature model.

Within SVM, we used the k-fold method for cross-validation (CV) with k = 10, which minimizes the risk of false positive features. This CV method randomly divides the data into k portions in which k − 1 portion is considered as training data and the other portion as testing data. By continuing this k-times, all subjects in the data set are part of both the training and testing set. The resulting classification accuracy is the average of all k-folds^63^. SVM analyses were performed in Python^64^ using the Scikit-learn package^65^. More details on our SVM analyses (e.g., feature selection method, statistical approach) can be found in the Supplement.

## Data Availability

The datasets generated and analyzed during the current study are not publicly available due to medical confidentiality but are available from the corresponding author on reasonable request. However, the used code is available under URL (https://github.com/erikelster/svm_asd_adhd.git). As the original data that was used for the presented results cannot be published as they contain clinical information, sample data is provided.
